# A Report of Cases Treated with Gonococcus Vaccine

**Published:** 1912-05

**Authors:** J. G. W. Knoll

**Affiliations:** Buffalo, N. Y. 482 William Street


### A Report of Cases Treated With Gonococcus Vaccine.

By J. G. W. KNOLL, M.D.
Buffalo, N. Y.

CASE No. 1. Woman age 27. Before coming under my observation had both tubes removed, but when she first consulted me there was a greenish yellow discharge from the uterus; microscopic examinaion showed gonococci, she was weak, anemic, and complained of pains all over the body. There was every evidence of a general systemic gonorrheal infection. I administered 40,000,000 Gonococcus Vaccine (P. D. & Co.) which produced a severe reaction with malaise and general increase of unfavorable symptoms so that she was confined to bed for four days. Seven days after the date of first injection, I gave 20,000,000 and this was also followed by a severe reaction, which kept her in bed three days. Seven days later I gave 10,000,000 with no reaction. No more vaccine was given, but the discharge and other symptoms all disappeared, and she has remained well to date.

*Case No. 2.* Man, age 31. History of gonorrhea five years ago, two years ago had an attack of gonorrheal rheumatism in hip, knees and wrist, which laid him up for three months. Before he had entirely recovered from the first attack there was recurrence of gonorrheal rheumatism in both knees and right hip; the greenish yellow urethral discharge showed gonococci. I gave 20,000,000 Gonoccus Vaccine (P. D. & Co.) no reaction. Five days later, gave 40,000,000; still no reaction. The discharge ceased, pain left right side in both hip and knee, and swelling disappeared. Joint on left side softened, and the pain lessened. Five days later, gave 100,000,000 Gonococcus Vaccine; still no reaction and all symptoms disappeared. Seven days later gave 200,000,000 Gonoccus Vaccine; patient had severe reaction, pain and soreness in all previously affected joints, but no discharge. Miscropic examination of urine showed no gonococci. Two weeks later gave 100,000,000 Gonococcus Vaccine, without any reaction. Patient was discharged cured. Have kept him under observation since, but he has shown no return of symptoms.

*Case No. 3.* Gonorrheal Orchitis, 48 hours standing. Gave one dose of 40,000,000 Gonococcus Vaccine (P. D. & Co.) and all symptoms disappeared in three days.

I have cured several cases of "morning drop" by administering three doses of 50,000,000 Gonococcus Vaccine each with a seven day interval between doses; it has never been necessary to give more.

482 WILLIAM STREET.

